# Implementation of a Toffoli gate using an array of coupled cavities in a single step

**DOI:** 10.1038/s41598-018-24214-4

**Published:** 2018-04-11

**Authors:** Y. Cao, G. C. Wang, H. D. Liu, C. F. Sun

**Affiliations:** 1 Center for Quantum Sciences and School of Physics, Northeast Normal University, Changchun, 130024 China; 2 Center for Advanced Optoelectronic Functional Materials Research, and Key Laboratory for UV Light-Emitting Materials and Technology of Ministry of Education, Northeast Normal University, Changchun, 130024 China

## Abstract

The Toffoli gate (controlled-controlled-NOT gate) is one typical three-qubit gate, it plus a Hadamard gate form a universal set of gates in quantum computation. We present an efficient method to implement the Toffoli gate using an array of coupled cavities with one three-level atom in each cavity. The large detuning between atoms and classical (quantum) fields plays an important role and the gate is implemented in one-step. The quantum information is encoded into the low-lying states of identical atoms and it is convenient to address qubit individually. Based on the Markovian master equation, it is shown that the scheme to implement the Toffoli gate is robust against the decoherence.

## Introduction

Quantum computers provide the possibility of solving certain computational tasks much faster than any classical counterpart using the best currently known algorithms^1–5^, thus a great deal of effort has been devoted to building scalable and functional quantum computers over the last two decades. Solving a quantum computational task corresponds to performing a unitary transformation on the quantum register, which is composed of multiple qubits. Any quantum algorithm can be decomposed into a sequence of single-qubit rotations and entangling two-qubit logical gates, which form a universal set of quantum operations^6,7^. Moreover, various kinds of physical realizations of quantum computations have been intensively studied^8–15^. However, if only single-qubit and two-qubit gates are available, the qubits scale up so that the approach becomes very complicated and it may be hard to implement. On the other hand, using gates acting on more than two qubits can significantly simplify the decomposition of otherwise intractable algorithms£¬ which can shorten the operation time and promise higher fidelity. Therefore multiqubit gates play a central role in quantum algorithms, quantum corrections, and quantum networks, and they serve as a stepping stone towards the realization of a scalable quantum computer.

Among the multiqubit gates, the quantum Toffoli gate (controlled-controlled-NOT gate)^16^ is one typical three-qubit gate. It flips the state of a target qubit conditioned on the state of two control qubits. This gate plus a Hadamard gate can form a universal set of gates in quantum computation. Moreover, it can be directly used to implement the complex quantum algorithms^1^ and quantum simulation^17–19^, and has immediate practical applications as correcting operation in quantum error correction schemes^20–24^. Therefore, the Toffoli gate is vitally important for quantum computing, and improving the design of the Toffoli gate can make the implementation of large-scale quantum computation more tractable. So far, the minimum cost for implementing a three-qubit Toffoli gate is five two-qubit gates^25^, and the decomposition of a generalized *n*-qubit Toffoli gate involves *O*(*n*^2^) two-qubit gates^7^. In experiment the Toffoli gate has been first implemented in nuclear magnetic resonance^20^. Recently, the three-qubit quantum Toffoli gate has been achieved in some other physical architectures, such as linear optics, ion-trap qubits, superconducting circuits, quantum dots (QDs), and diamond nitrogen-vacancy (NV) defect centres^26–32^. In these experiments based on the idea of “hiding” states, the required resources are greatly reduced in contrast with theoretical proposals, which use only two-level systems. However, they still require three two-qubit or qubit-qutrit gates so that the fidelity would be significantly deteriorated by decoherence. Therefore, it is important to implement the quantum Toffoli gate in one-step without using any two-qubit or qubit-qutrit gate.

In this work, we propose an efficient scheme to realize a Toffoli gate in one-step with a coupled-cavity model. The matrix form of the three-qubit Toffoli gate expanded in the basis {|0〉_1_, |1〉_1_, |0〉_2_, |1〉_2_, |0〉_3_, |1〉_3_} is

1 $${U}_{{\rm{toff}}}=(\begin{array}{llllllll}1 & 0 & 0 & 0 & 0 & 0 & 0 & 0\\ 0 & 1 & 0 & 0 & 0 & 0 & 0 & 0\\ 0 & 0 & 0 & 1 & 0 & 0 & 0 & 0\\ 0 & 0 & 1 & 0 & 0 & 0 & 0 & 0\\ 0 & 0 & 0 & 0 & 1 & 0 & 0 & 0\\ 0 & 0 & 0 & 0 & 0 & 1 & 0 & 0\\ 0 & 0 & 0 & 0 & 0 & 0 & 1 & 0\\ 0 & 0 & 0 & 0 & 0 & 0 & 0 & 1\end{array}),$$

where the target qubit swaps its information $\mathrm{|0}{\rangle }_{3}\iff \mathrm{|1}{\rangle }_{3}$ if and only if two control qubits are in |01〉_12_. Note that it is equivalent to the standard form of a Toffoli gate upon a local unitary transformation. Coupled cavity arrays describes a series of optical cavities, each of which contains one or more qubits or atoms, and photons can hop between two neighboring cavities. This model can overcome the problem of individual addressability and has emerged as a fascinating alternative for simulating quantum many-body phenomena. Theoretical works on quantum information processing and quantum computing have been proposed with using the atom-cavity interaction in coupled cavity arrays^33–41^. The merit of our scheme is that the Toffoli gate is implemented in one-step without any single-qubit or two-qubit operation, which can significantly simplify the experimental realization and shorten the operation time. Meanwhile, it is easy to control and measure qubit separately because there is one three-level atom in each cavity. Furthermore, we encode the quantum information into the low-lying states of three identical atoms without any ancillary level compared with ref.^42^.

## Results

**Figure 1:**
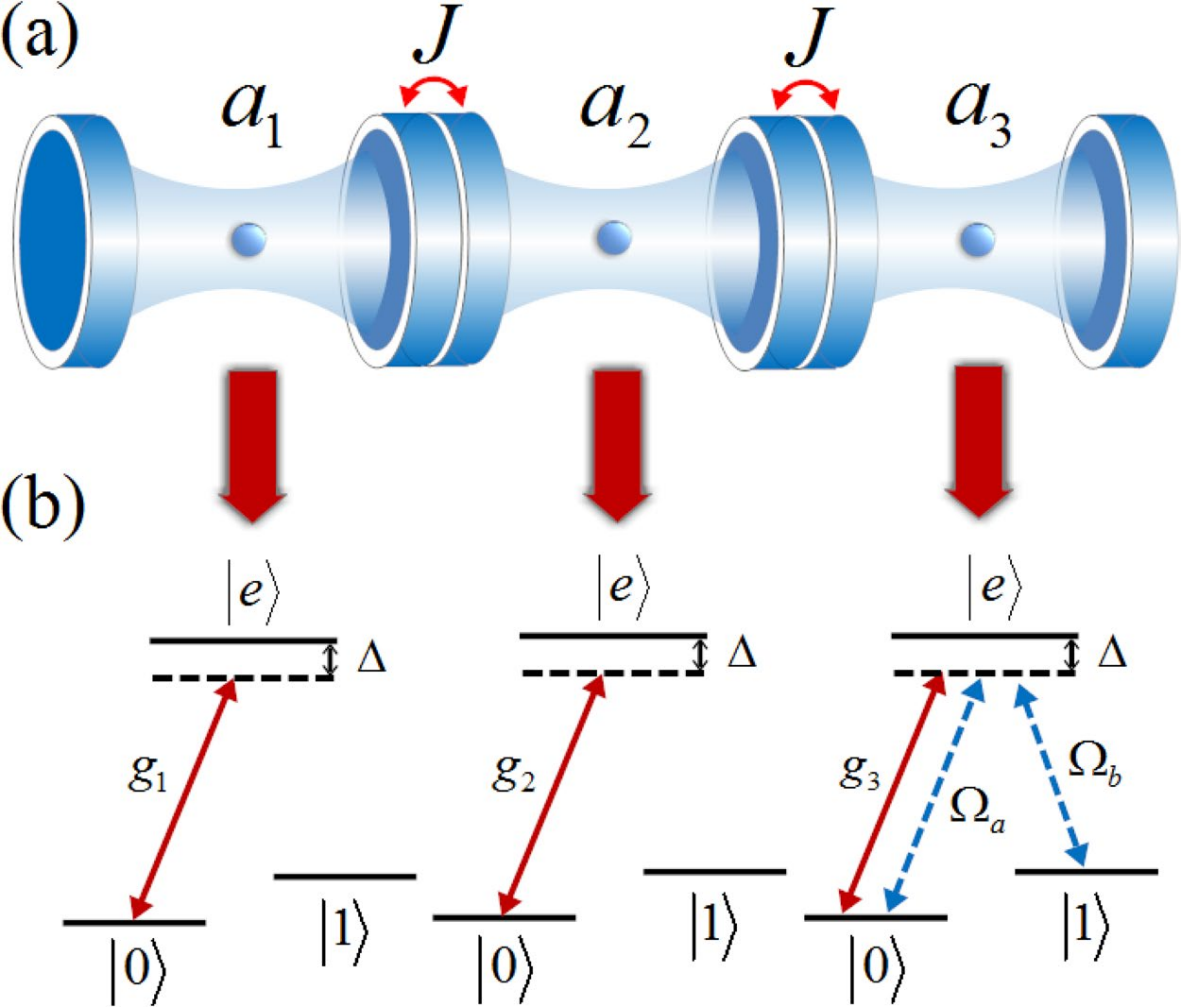
Three coupled cavities with one three-level atom in each cavity are shown for simulating the Toffoli gate. From left to right the atoms are labeled as 1, 2, 3. We consider the first two atoms as the control qubits while the third atom is the target qubit. The two ground state levels |0_*i*_〉 and |1_*i*_〉 (*i* = 1, 2, 3) of each atom define a single qubit.

In Fig. 1 we consider three coupled cavities with one three-level atom in each cavity. The *k*-th (*k* = 1, 2, 3) atom has two ground states |0_*k*_〉 and |1_*k*_〉 and one excited state |*e*_*k*_〉 with energies *ω*_*a*_, *ω*_*b*_ and *ω*_*e*_, respectively. Each ${\mathrm{|0}}_{k}\rangle \leftrightarrow |{e}_{k}\rangle$ transition is coupled to its corresponding cavity mode with the coupling strength *g*_*k*_, detuned by Δ. Meanwhile, the transitions ${\mathrm{|0}}_{3}\rangle \leftrightarrow |{e}_{3}\rangle$ and ${\mathrm{|1}}_{3}\rangle \leftrightarrow |{e}_{3}\rangle$ for the target atom are driven by a pair of classical fields with the Rabi frequencies Ω_*a*_ and Ω_*b*_ respectively, detuned by the same parameter Δ. In addition, the cavities are coupled via the exchange of photons with the coupling constant *J*. The system Hamiltonian takes the following form (*ħ* = 1)

2 $$H={H}_{{\rm{cla}}}+{H}_{{\rm{int}}}+{H}_{{\rm{hop}}}+{H}_{{\rm{free}}},$$

where

3 $$\begin{array}{rcl}{H}_{{\rm{cla}}} & = & {{\rm{\Omega }}}_{a}(|{e}_{3}\rangle \langle {0}_{3}|{e}^{-i{\omega }_{{l}_{1}}t}+{\mathrm{|0}}_{3}\rangle \langle {e}_{3}|{e}^{i{\omega }_{{l}_{1}}t})\\  &  & +{{\rm{\Omega }}}_{b}(|{e}_{3}\rangle \langle {1}_{3}|{e}^{-i{\omega }_{{l}_{2}}t}+{\mathrm{|1}}_{3}\rangle \langle {e}_{3}|{e}^{i{\omega }_{{l}_{2}}t}),\\ {H}_{{\rm{int}}} & = & \sum _{k=1}^{3}\,{g}_{k}({a}_{k}|{e}_{k}\rangle \langle {0}_{k}|+{a}_{k}^{\dagger }{\mathrm{|0}}_{k}\rangle \langle {e}_{k}|),\\ {H}_{{\rm{hop}}} & = & \sum _{j=1}^{2}\,J({a}_{j}^{\dagger }{a}_{j+1}+{a}_{j}{a}_{j+1}^{\dagger }),\\ {H}_{{\rm{free}}} & = & \sum _{k=1}^{3}\,({\omega }_{e}|{e}_{k}\rangle \langle {e}_{k}|+{\omega }_{a}{\mathrm{|0}}_{k}\rangle \langle {0}_{k}|+{\omega }_{b}{\mathrm{|1}}_{k}\rangle \langle {1}_{k}|)+\sum _{k=1}^{3}\,{\omega }_{c}{a}_{k}^{\dagger }{a}_{k},\end{array}$$

where ${a}_{j}({a}_{j}^{\dagger })$ is the annihilation (creation) operator of the j-th cavity mode, ${\omega }_{{l}_{1}}$ and ${\omega }_{{l}_{2}}$ are the frequencies of two classical fields, and *ω*_*c*_ is the frequency of the cavity. By changing to the interaction picture, and performing a rotation with the frame defined by $U=\exp (i{\rm{\Delta }}t\,{\sum }_{i=1}^{3}\,|{e}_{i}\rangle \langle {e}_{i}|)$, the Hamiltonian can be written as

4 $$\begin{array}{rcl}{H}_{I} & = & {{\rm{\Omega }}}_{a}(|{e}_{3}\rangle \langle {0}_{3}|+{\mathrm{|0}}_{3}\rangle \langle {e}_{3}|)+{{\rm{\Omega }}}_{b}(|{e}_{3}\rangle \langle {1}_{3}|+{\mathrm{|1}}_{3}\rangle \langle {e}_{3}|)\\  &  & +\sum _{k=1}^{3}\,{g}_{k}({a}_{k}|{e}_{k}\rangle \langle {0}_{k}|+{a}_{k}^{\dagger }{\mathrm{|0}}_{k}\rangle \langle {e}_{k}|)\\  &  & +\sum _{j=1}^{2}\,J({a}_{j}^{\dagger }{a}_{j+1}+{a}_{j}{a}_{j+1}^{\dagger })+\sum _{k=1}^{3}\,{\rm{\Delta }}|{e}_{k}\rangle \langle {e}_{k}|,\end{array}$$

where we have assumed *g*_*k*_ = *g* for simplicity.

In the following, we will discuss the scheme to implement the Toffoli gate based on the large detuning case. Here we consider that the two classical optical pumping lasers are both sufficiently weak (i.e. the Rabi frequencies Ω_*a*_ and Ω_*b*_ are both very small compared with {*J*, *g*, Δ}), and the excited states of the atoms and the excited cavity field modes are not initially populated, the highly excited level can be neglected^33–35,43,44^. Based on the interaction form of the Hamiltonian (4), the qubit basis {|0〉_1_, |1〉_1_, |0〉_2_, |1〉_2_, |0〉_3_, |1〉_3_}_*a*_ with cavities in vacuum states can be divided into four subspaces. For the first subspace {|010〉_*a*_ |000〉_*c*_, |011〉_*a*_ |000〉_*c*_, |*e*10〉_*a*_ |000〉_*c*_, |01*e*〉_*a*_ |000〉_*c*_, |010〉_*a*_ |100〉_*c*_, |010〉_*a*_ |010〉_*c*_, |010〉_*a*_ |001〉_*c*_}, we first diagonalize the strong interaction described by atom-cavity Hamiltonian in Eq. (4). Based on the new basis $\{|{{\rm{\Phi }}}_{1}^{\mathrm{(1)}}\rangle$, $|{{\rm{\Phi }}}_{2}^{\mathrm{(1)}}\rangle$, $|{{\rm{\Phi }}}_{a}^{\mathrm{(1)}}\rangle$, $|{{\rm{\Phi }}}_{b}^{\mathrm{(1)}}\rangle$, $|{{\rm{\Phi }}}_{3}^{\mathrm{(1)}}\rangle \}$ with

5 $$\begin{array}{rcl}|{{\rm{\Phi }}}_{1}^{\mathrm{(1)}}\rangle  & = & \frac{1}{2}\mathrm{|010}{\rangle }_{a}{(\mathrm{|100}\rangle -\sqrt{2}\mathrm{|010}\rangle +\mathrm{|001}\rangle )}_{c},\\ |{{\rm{\Phi }}}_{2}^{\mathrm{(1)}}\rangle  & = & \frac{1}{2}\mathrm{|010}{\rangle }_{a}{(\mathrm{|100}\rangle +\sqrt{2}\mathrm{|010}\rangle +\mathrm{|001}\rangle )}_{c},\\ |{{\rm{\Phi }}}_{3}^{\mathrm{(1)}}\rangle  & = & \frac{1}{\sqrt{2}}\mathrm{|010}{\rangle }_{a}\mathrm{(|100}\rangle -\mathrm{|001}\rangle {)}_{c},\\ |{{\rm{\Phi }}}_{a}^{\mathrm{(1)}}\rangle  & = & \frac{1}{\sqrt{2}}(|e10\rangle +\mathrm{|01}e\rangle {)}_{a}\mathrm{|000}{\rangle }_{c},\\ |{{\rm{\Phi }}}_{b}^{\mathrm{(1)}}\rangle  & = & \frac{1}{\sqrt{2}}(|e10\rangle -\mathrm{|01}e\rangle {)}_{a}\mathrm{|000}{\rangle }_{c},\end{array}$$

the atom-cavity Hamiltonian reads:

6 $${T}_{1}=(\begin{array}{ccccc}-\sqrt{2}J & 0 & \frac{g}{\sqrt{2}} & 0 & 0\\ 0 & \sqrt{2}J & \frac{g}{\sqrt{2}} & 0 & 0\\ \frac{g}{\sqrt{2}} & \frac{g}{\sqrt{2}} & {\rm{\Delta }} & 0 & 0\\ 0 & 0 & 0 & {\rm{\Delta }} & g\\ 0 & 0 & 0 & g & 0\end{array}).$$

The effective Hamiltonian in the first subspace can be evaluated explicitly (see Methods)

7 $$\begin{array}{rcl}{H}_{{\rm{eff}}}^{1} & = & -\frac{{{\rm{\Omega }}}_{a}^{2}}{2{\rm{\Delta }}}\mathrm{|010}\rangle \langle \mathrm{010|}-\frac{{{\rm{\Omega }}}_{b}^{2}}{2{\rm{\Delta }}}\mathrm{|011}\rangle \langle \mathrm{011|}\\  &  & -\frac{{{\rm{\Omega }}}_{a}{{\rm{\Omega }}}_{b}}{2{\rm{\Delta }}}\mathrm{(|011}\rangle \langle \mathrm{010|}+\mathrm{|010}\rangle \langle \mathrm{011|)}.\end{array}$$

Similar to the analysis of the first subspace, we consider the second subspace {|000〉_*a*_ |000〉_*c*_, |001〉_*a*_ |000〉_*c*_, |*e*00〉_*a*_ |000〉_*c*_, |0*e*0〉_*a*_ |000〉_*c*_, |00*e*〉_*a*_ |100〉_*c*_, |000〉_*a*_ |100〉_*c*_, |000〉_*a*_ |010〉_*c*_, |000〉_*a*_ |001〉_*c*_}. Based on the new basis $\{|{{\rm{\Phi }}}_{1}^{\mathrm{(2)}}\rangle$, $|{{\rm{\Phi }}}_{a}^{\mathrm{(2)}}\rangle$, $|{{\rm{\Phi }}}_{2}^{\mathrm{(2)}}\rangle$, $|{{\rm{\Phi }}}_{b}^{\mathrm{(2)}}\rangle$, $|{{\rm{\Phi }}}_{3}^{\mathrm{(2)}}\rangle$, $|{{\rm{\Phi }}}_{c}^{\mathrm{(2)}}\rangle \}$ with

8 $$\begin{array}{rcl}|{{\rm{\Phi }}}_{1}^{\mathrm{(2)}}\rangle  & = & \frac{1}{2}\mathrm{|000}{\rangle }_{a}{(\mathrm{|100}\rangle -\sqrt{2}\mathrm{|010}\rangle +\mathrm{|001}\rangle )}_{c},\\ |{{\rm{\Phi }}}_{2}^{\mathrm{(2)}}\rangle  & = & \frac{1}{2}\mathrm{|000}{\rangle }_{a}{(\mathrm{|100}\rangle +\sqrt{2}\mathrm{|010}\rangle +\mathrm{|001}\rangle )}_{c},\\ |{{\rm{\Phi }}}_{3}^{\mathrm{(2)}}\rangle  & = & \frac{1}{\sqrt{2}}\mathrm{|000}{\rangle }_{a}\mathrm{(|100}\rangle -\mathrm{|001}\rangle {)}_{c},\\ |{{\rm{\Phi }}}_{a}^{\mathrm{(2)}}\rangle  & = & \frac{1}{2}{(|e00\rangle -\sqrt{2}\mathrm{|0}e0\rangle +\mathrm{|00}e\rangle )}_{a}\mathrm{|000}{\rangle }_{c},\\ |{{\rm{\Phi }}}_{b}^{\mathrm{(2)}}\rangle  & = & \frac{1}{2}{(|e00\rangle +\sqrt{2}\mathrm{|0}e0\rangle +\mathrm{|00}e\rangle )}_{a}\mathrm{|000}{\rangle }_{c},\\ |{{\rm{\Phi }}}_{c}^{\mathrm{(2)}}\rangle  & = & \frac{1}{\sqrt{2}}(|e00\rangle -\mathrm{|00}e\rangle {)}_{a}\mathrm{|000}{\rangle }_{c},\end{array}$$

the atom-cavity Hamiltonian reads:

9 $${T}_{2}=(\begin{array}{cccccc}-\sqrt{2}J & g & 0 & 0 & 0 & 0\\ g & {\rm{\Delta }} & 0 & 0 & 0 & 0\\ 0 & 0 & \sqrt{2}J & g & 0 & 0\\ 0 & 0 & g & {\rm{\Delta }} & 0 & 0\\ 0 & 0 & 0 & 0 & 0 & g\\ 0 & 0 & 0 & 0 & g & {\rm{\Delta }}\end{array}).$$

The effective Hamiltonian in the second subspace reduces to (see Methods)

10 $$\begin{array}{rcl}{H}_{{\rm{eff}}}^{2} & = & \frac{1}{\frac{{g}^{4}}{{J}^{2}{\rm{\Delta }}}-2{\rm{\Delta }}}[{{\rm{\Omega }}}_{a}^{2}\mathrm{|000}\rangle \langle \mathrm{000|}+{{\rm{\Omega }}}_{b}^{2}\mathrm{|001}\rangle \langle \mathrm{001|}\\  &  & +{{\rm{\Omega }}}_{a}{{\rm{\Omega }}}_{b}\mathrm{(|000}\rangle \langle \mathrm{001|}+\mathrm{|100}\rangle \langle \mathrm{000|)]}.\end{array}$$

Based on the above condition $\{|{{\rm{\Omega }}}_{a}|,|{{\rm{\Omega }}}_{b}|\}\ll \{J,g,{\rm{\Delta }}\}$ and $J\ll g$, this effective Hamiltonian is ${H}_{{\rm{eff}}}^{2}\approx 0$, which means that the qubit states |000〉 and |001〉 remain unchanged during the whole evolution time.

For the third subspace {|100〉_*a*_ |000〉_*c*_, |101〉_*a*_ |000〉_*c*_, |1*e*0〉_*a*_ |000〉_*c*_, |10*e*〉_*a*_ |000〉_*c*_, |100〉_*a*_ |100〉_*c*_, |100〉_*a*_ |010〉_*c*_, |100〉_*a*_ |001〉_*c*_}, based on the new basis $\{|{{\rm{\Phi }}}_{1}^{\mathrm{(3)}}\rangle$, $|{{\rm{\Phi }}}_{2}^{\mathrm{(3)}}\rangle$, $|{{\rm{\Phi }}}_{3}^{\mathrm{(3)}}\rangle$, $|{{\rm{\Phi }}}_{a}^{\mathrm{(3)}}\rangle$, $|{{\rm{\Phi }}}_{b}^{\mathrm{(3)}}\rangle \}$ with

11 $$\begin{array}{rcl}|{{\rm{\Phi }}}_{1}^{\mathrm{(3)}}\rangle  & = & \frac{1}{2}\mathrm{|100}{\rangle }_{a}{(\mathrm{|100}\rangle -\sqrt{2}\mathrm{|010}\rangle +\mathrm{|001}\rangle )}_{c},\\ |{{\rm{\Phi }}}_{2}^{\mathrm{(3)}}\rangle  & = & \frac{1}{2}\mathrm{|100}{\rangle }_{a}{(\mathrm{|100}\rangle +\sqrt{2}\mathrm{|010}\rangle +\mathrm{|001}\rangle )}_{c},\\ |{{\rm{\Phi }}}_{3}^{\mathrm{(3)}}\rangle  & = & \frac{1}{\sqrt{2}}\mathrm{|100}{\rangle }_{a}\mathrm{(|100}\rangle -\mathrm{|001}\rangle {)}_{c},\\ |{{\rm{\Phi }}}_{a}^{\mathrm{(3)}}\rangle  & = & \frac{1}{\sqrt{2}}\mathrm{(|1}e0\rangle +\mathrm{|10}e\rangle {)}_{a}\mathrm{|000}{\rangle }_{c},\\ |{{\rm{\Phi }}}_{b}^{\mathrm{(3)}}\rangle  & = & \frac{1}{\sqrt{2}}\mathrm{(|1}e0\rangle -\mathrm{|10}e\rangle {)}_{a}\mathrm{|000}{\rangle }_{c},\end{array}$$

the atom-cavity Hamiltonian is given by:

12 $${T}_{3}=(\begin{array}{ccccc}-\sqrt{2}J & 0 & 0 & -\frac{2-\sqrt{2}}{4}g & -\frac{2+\sqrt{2}}{4}g\\ 0 & \sqrt{2}J & 0 & \frac{2+\sqrt{2}}{4}g & \frac{2-\sqrt{2}}{4}g\\ 0 & 0 & 0 & -\frac{g}{2} & \frac{g}{2}\\ -\frac{2-\sqrt{2}}{4}g & \frac{2+\sqrt{2}}{4}g & -\frac{g}{2} & {\rm{\Delta }} & 0\\ -\frac{2+\sqrt{2}}{4}g & \frac{2-\sqrt{2}}{4}g & \frac{g}{2} & 0 & {\rm{\Delta }}\end{array}).$$

The effective Hamiltonian in the third subspace is (see Methods)

13 $${H}_{{\rm{eff}}}^{3}=0,$$

which means that the qubit states |100〉 and |101〉 remain unchanged during the whole evolution time.

For the fourth subspace {|110〉_*a*_ |000〉_*c*_, |111〉_*a*_ |000〉_*c*_, |11*e*〉_*a*_ |000〉_*c*_, |110〉_*a*_ |100〉_*c*_, |110〉_*a*_ |010〉_*c*_, |110〉_*a*_ |001〉_*c*_}, based on the new basis $\{|{{\rm{\Phi }}}_{1}^{\mathrm{(4)}}\rangle$, $|{{\rm{\Phi }}}_{2}^{\mathrm{(4)}}\rangle$, $|{{\rm{\Phi }}}_{3}^{\mathrm{(4)}}\rangle$, $|{{\rm{\Phi }}}_{a}^{\mathrm{(4)}}\rangle \}$ with

14 $$\begin{array}{rcl}|{{\rm{\Phi }}}_{1}^{\mathrm{(4)}}\rangle  & = & \frac{1}{2}\mathrm{|110}{\rangle }_{a}{(\mathrm{|100}\rangle -\sqrt{2}\mathrm{|010}\rangle +\mathrm{|001}\rangle )}_{c},\\ |{{\rm{\Phi }}}_{2}^{\mathrm{(4)}}\rangle  & = & \frac{1}{2}\mathrm{|110}{\rangle }_{a}{(\mathrm{|100}\rangle +\sqrt{2}\mathrm{|010}\rangle +\mathrm{|001}\rangle )}_{c},\\ |{{\rm{\Phi }}}_{3}^{\mathrm{(4)}}\rangle  & = & \frac{1}{\sqrt{2}}\mathrm{|110}{\rangle }_{a}\mathrm{(|100}\rangle -\mathrm{|001}\rangle {)}_{c},\\ |{{\rm{\Phi }}}_{a}^{\mathrm{(4)}}\rangle  & = & \mathrm{|11}e{\rangle }_{a}\mathrm{|000}{\rangle }_{c},\end{array}$$

the atom-cavity Hamiltonian reads:

15 $${T}_{4}=(\begin{array}{cccc}-\sqrt{2}J & 0 & 0 & \frac{g}{2}\\ 0 & \sqrt{2}J & 0 & \frac{g}{2}\\ 0 & 0 & 0 & -\frac{g}{\sqrt{2}}\\ \frac{g}{2} & \frac{g}{2} & -\frac{g}{\sqrt{2}} & {\rm{\Delta }}\end{array}).$$

The effective Hamiltonian in the fourth subspace is (see Methods)

16 $${H}_{{\rm{eff}}}^{4}=\mathrm{0,}$$

which means that the qubit states |110〉 and |111〉 remain unchanged during the whole evolution time. Thus the time evolution operator for the final effective Hamiltonian can be written as

17 $$\begin{array}{rcl}U(t) & = & \frac{1}{2}\mathrm{(1}-\exp (imt))\,\mathrm{(|010}\rangle \langle \mathrm{011|}+\mathrm{|011}\rangle \langle \mathrm{010|)}\\  &  & +\frac{1}{2}\mathrm{(1}+\exp (imt))\,\mathrm{(|010}\rangle \langle \mathrm{010|}+\mathrm{|011}\rangle \langle \mathrm{011|),}\end{array}$$

where *m* = Ω^2^/Δ with the parameters Ω_*b*_ = −Ω_*a*_ = Ω. Adjust the evolution period *T* = *π*Δ/Ω^2^, we obtain the three-qubit Toffoli gate which takes the form of Eq. (1).

**Figure 2:**
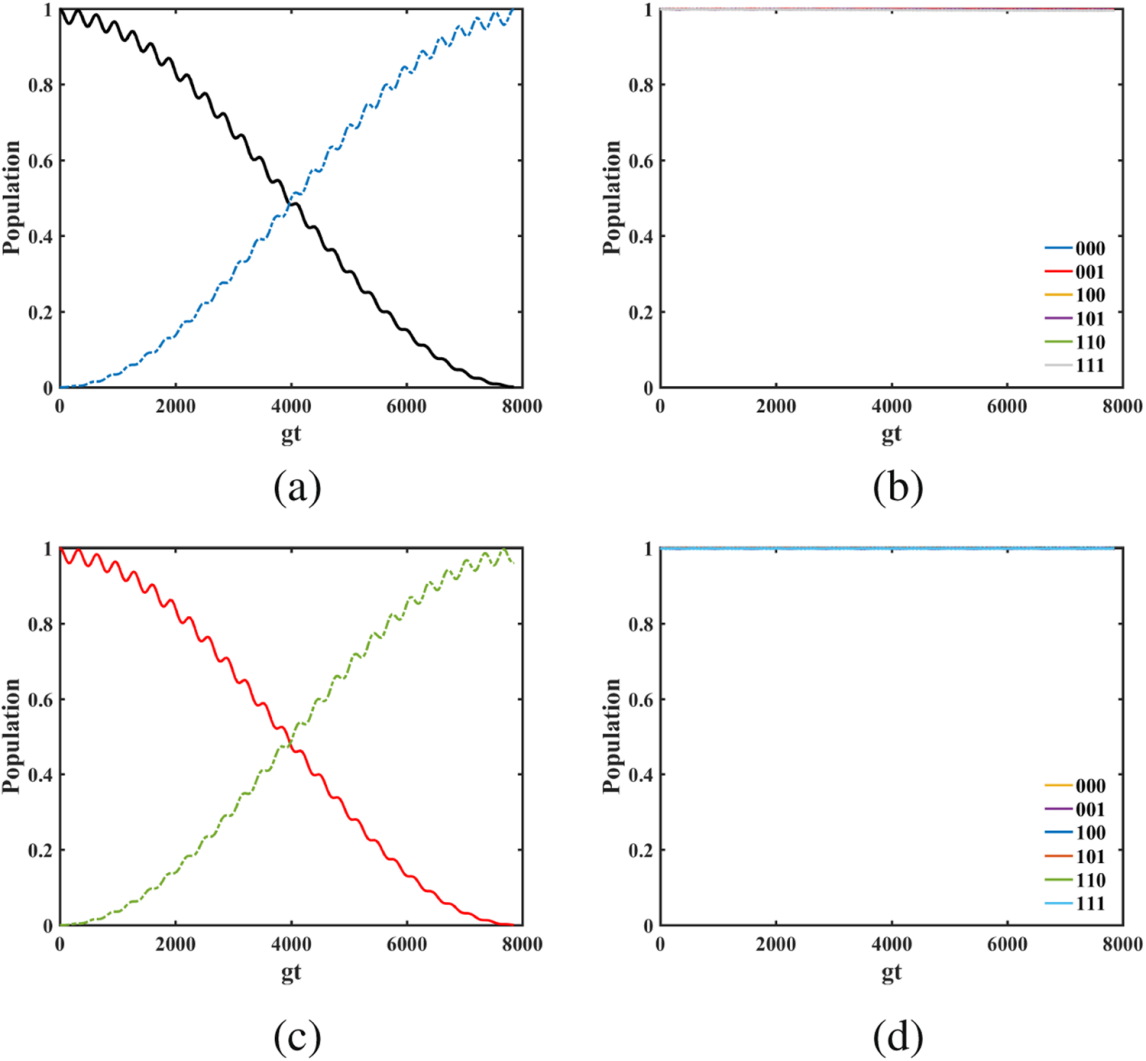
The populations of three qubit states when the system reserves the single excitation (**a**,**b**) and the double excitation (**c**,**d**), respectively. (**a**,**c**) Plot the populations conversion of states |010〉 and |011〉; (**b**,**d**) Plot the populations of states |000〉, |001〉, |100〉, |101〉, |110〉 and |111〉. The excited cavity field modes can be adiabatically eliminated. Choose the parameters as Ω_*b*_ = −Ω_*a*_ = Ω = 0.02*g*, Δ = *g* and *J* = 0.1*g*.

**Figure 3:**
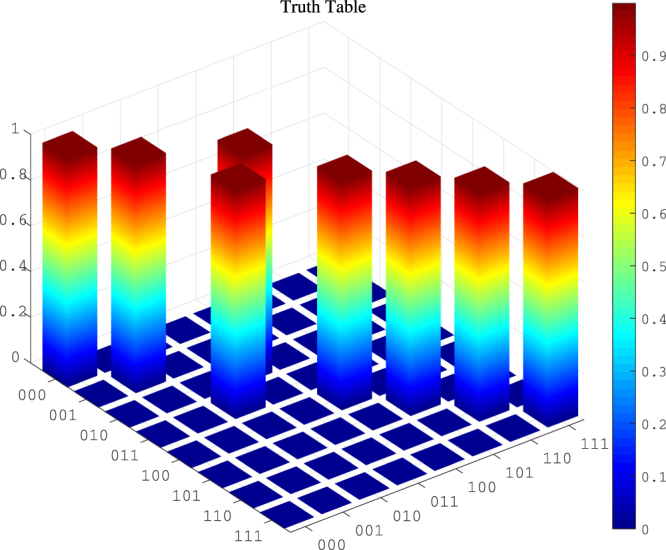
The truth table of the population of the Toffoli gate at the period time for the single excitation case. The parameters are chosen as Ω_*b*_ = −Ω_*a*_ = Ω = 0.02*g*, Δ = *g* and *J* = 0.1*g*.

**Figure 4:**
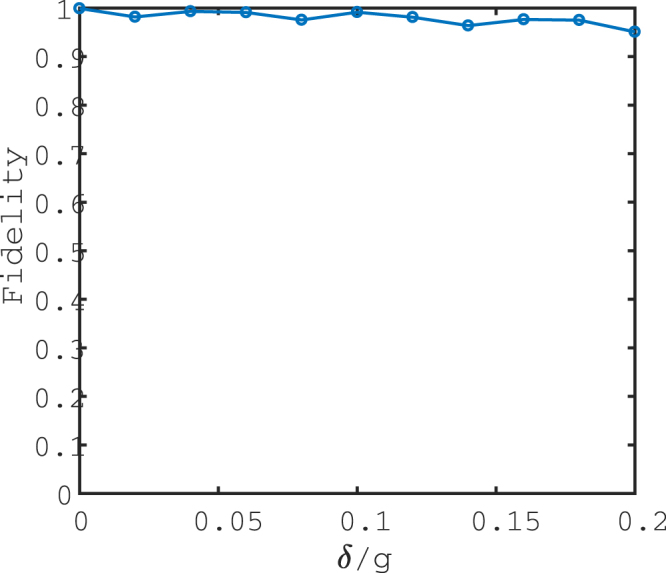
The fidelity *F* of the Toffoli gate versus the parameter *δ*/*g*. The atom-cavity coupling constants *g*_1_ = *g* + *δ*, *g*_2_ = *g* − *δ* and *g*_3_ = *g*. The parameters are chosen as Ω_*b*_ = −Ω_*a*_ = Ω = 0.02*g*, Δ = *g* and *J* = 0.1*g*.

In what follows, we check the accuracy of the effective Hamiltonian compared to the original Hamiltonian with the populations of three qubit states {|000〉, |001〉, |010〉, |011〉, |100〉, |101〉, |110〉, |111〉}_*a*_ |000〉_*c*_. In Fig. 2, (a) plots the conversion of states |010〉_*a*_ |000〉_*c*_ and |011〉_*a*_ |000〉_*c*_ when the system reserves the single excitation. The population can achieve 0.9981 at the period time. (b) depicts the populations of states |000〉_*a*_ |000〉_*c*_, |001〉_*a*_ |000〉_*c*_, |100〉_*a*_ |000〉_*c*_, |101〉_*a*_ |000〉_*c*_, |110〉_*a*_ |000〉_*c*_ and |111〉_*a*_ |000〉_*c*_ for the system reserving the single excitation. The minimum data of the population is 0.9938 during the evolution time. Since the system do not conserve the total number of excitations, and in front we neglect the highly excited level under the weak excitation case, here we further consider the numerical simulation for the system reserving the double excitation in (c) and (d). Compare plots (c) with (a), (d) with (b), it is found that the results for the double excitation case are in accord with the results for the single excitation case. These numerical results reveal that the effective Hamiltonian is excellently close to the original Hamiltonian under the given parameters. To make our results more clearly, Fig. 3 gives the truth table of the Toffoli gate at the period time for the single excitation case. The fidelity for the Toffoli gate in the ideal case is $F(T)=\frac{1}{8}|{\rm{tr}}[{U}^{\dagger }(T){U}_{{\rm{Toffoli}}}]|=0.9991$, with *U*(*T*) being the final evolution operator based on the original Hamiltonian (4) and *U*_Toffoli_ being the ideal Toffoli gate. Thus a Toffoli gate is implemented with high fidelity. Furthermore, we numerically discuss the case that the atom-cavity coupling strengths *g*_1_, *g*_2_ and *g*_3_ are different with *g*_1_ = *g* + *δ*, *g*_2_ = *g* − *δ* and *g*_3_ = *g*, the Toffili gate can be implemented as well, as shown in Fig. 4. When the parameter *δ* ≤ 0.2, the fidelity contains higher than 95%.

## Discussion

In the coupled-cavity arrays, the main decoherence effects in our scheme are the decay of cavities and the spontaneous emission of atoms. In this section, we numerically show how the decay of cavities and the spontaneous emission of atoms affect the fidelity of the resulting gate. The master equation for the whole system in the Markov approximation is governed by the following Lindblad equation^45^:

18 $$\begin{array}{rcl}\dot{\rho } & = & -i[{H}_{I},\rho ]+\sum _{j=1}^{3}\,\kappa ({a}_{j}\rho {a}_{j}^{\dagger }-\frac{1}{2}{a}_{j}^{\dagger }{a}_{j}\rho -\frac{1}{2}\rho {a}_{j}^{\dagger }{a}_{j})\\  &  & +\sum _{l=\mathrm{0,1}}\,\sum _{j=1}^{3}\,{\gamma }_{j}^{el}({\sigma }_{le}^{j}\rho {\sigma }_{el}^{j}-\frac{1}{2}{\sigma }_{ee}^{j}\rho -\frac{1}{2}\rho {\sigma }_{ee}^{j}),\end{array}$$

where *κ* represents the cavity decay rate, ${\gamma }_{j}^{el}$ denotes the spontaneous emission rate of atoms from the level |*e*〉_*j*_ to |*l*〉_*j*_ for the j-th atom (*j* = 1, 2, 3) and we assume ${\gamma }_{j}^{e0}={\gamma }_{j}^{e1}=\gamma /2$ for convenience. To quantify the robustness of our logical gate, we adopt the gate fidelity defined as the Bures-Uhlmann fidelity

19 $$F({\rho }_{id},\rho (t))\equiv {\rm{Tr}}\sqrt{{\rho }_{id}^{\frac{1}{2}}\rho (t){\rho }_{id}^{\frac{1}{2}}},$$

where *ρ*(*t*) is the mixed output system state (obtained from the joint system-bath evolution after a partial trace over the bath) and *ρ*_*id*_ is the density operator for target state. Here we choose the initial state as $$|{\rm{\Psi }}\rangle =\tfrac{1}{\sqrt{8}}\mathrm{(|000}\rangle +\mathrm{|001}\rangle +\mathrm{|010}\rangle -\mathrm{|011}\rangle +\mathrm{|100}\rangle +\mathrm{|101}\rangle +\mathrm{|110}\rangle +\mathrm{|111}\rangle {)}_{a}\mathrm{|000}{\rangle }_{c}$$. The corresponding density operator for target state is $${\rho }_{id}=|{\rm{\Psi }}^{\prime} \rangle \langle {\rm{\Psi }}^{\prime} |$$, with the target state $$|{\rm{\Psi }}^{\prime} \rangle =\tfrac{1}{\sqrt{8}}(|000\rangle +|001\rangle -|010\rangle +|011\rangle +$$$$|100\rangle +|101\rangle +|110\rangle +|111\rangle {)}_{a}|000{\rangle }_{c}$$.

**Figure 5:**
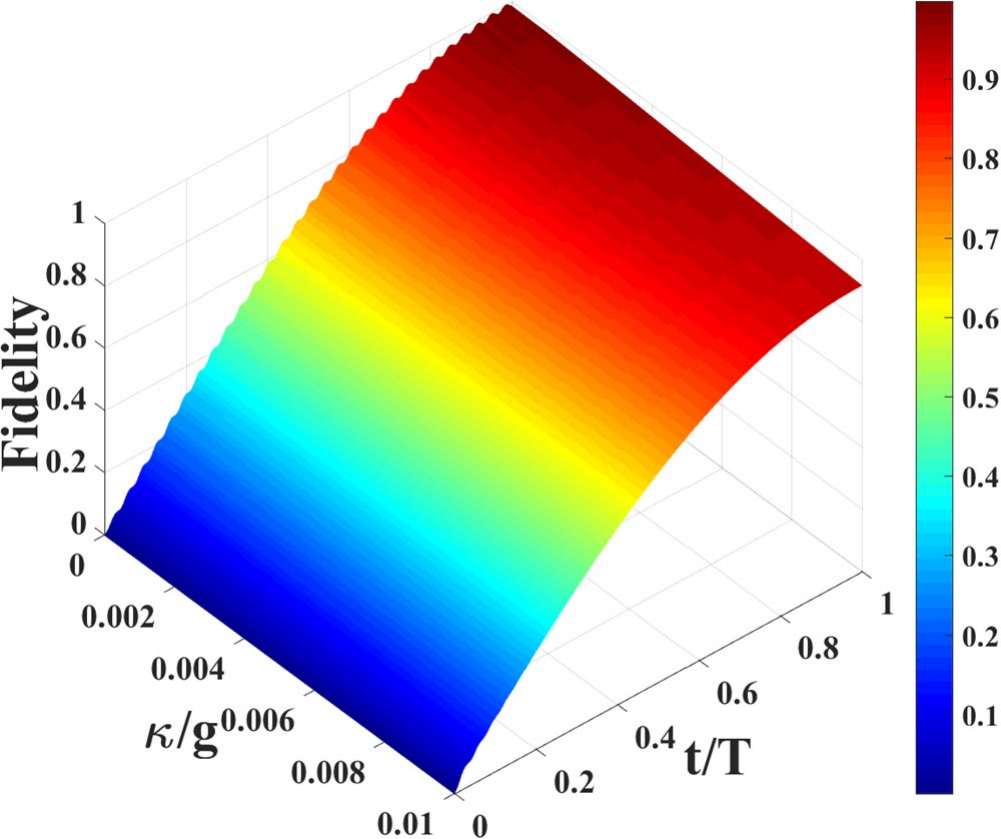
The fidelity *F* of the Toffoli gate versus the decoherence parameter *κ*/*g* and interaction time *t*/*T*, where *γ* = *κ*. The parameters are chosen as Ω_*b*_ = −Ω_*a*_ = Ω = 0.035*g*, Δ = *g* and *J* = 0.2*g*.

In Fig. 5 we depict the fidelity *F* of the Toffoli gate for the large detuning model as a function of the decoherence parameter *κ*/*g* and interaction time *t*/*T*. The fidelity *F* remains higher than 91%, which shows the Toffoli gate is robust against decoherence. Recently, the coupled cavity arrays can be constructed in several kinds of physical systems, such as photonic crystal defects^46^, toroidal microcavity arrays^47^, and superconducting stripline resonators^48^. Ref.^47^ investigated the suitability of toroidal microcavities for strong-coupling cavity quantum electrodynamics with the parameters $g\sim 2\pi \times 750$ MHz, $\gamma \sim 2\pi \times 2.62$ MHz, $\kappa \sim 2\pi \times 3.5$ MHz. And ref.^49^ has shown the large-scale ultrahigh-Q coupled nanocavity arrays based on photonic crystals corresponding to the parameters $g\sim 2.5\times {10}^{9}$ Hz, $\gamma \sim 1.6\times {10}^{7}$ Hz, $\kappa \sim 4\times {10}^{5}$ Hz. The fidelity of the Toffoli gate can achieve 95.43% and 98.14% for the above two different kinds of parameters (*g*, *γ*, *κ*), respectively. In the multi-qubit quantum computing networks the fidelities are relatively high.

In summary, we have proposed an efficient method to implement the Toffoli gate using an array of coupled cavities with one three-level atom in each cavity. The large detuning between atoms and classical (quantum) fields plays an important role. The Toffoli gate is implemented in one-step without any single-qubit or two-qubit operation, which can significantly simplify the experimental realization and shorten the operation time. Meanwhile, it is easy to control and measure qubit separately because there is one three-level atom in each cavity. Furthermore, we encode the quantum information into the low-lying states of three identical atoms without any ancillary level.

## Methods

### The effective Hamiltonian in the first subspace

In the case that the two classical optical pumping lasers are both sufficiently weak (i.e. the Rabi frequencies Ω_*a*_ and Ω_*b*_ are both very small compared with {*J*, *g*, Δ}), and the excited states are not initially populated, the excited states of the atoms and the excited cavity field modes can be adiabatically eliminated. The resulting effective dynamics will describe three two-level systems. To second order in perturbation theory, the dynamics are then given by the effective operators^44^:

20 $${H}_{{\rm{eff}}}=-\,\frac{1}{2}[{V}_{-}{H}_{NH}^{-1}{V}_{+}+{V}_{-}{({H}_{NH}^{-1})}^{\dagger }{V}_{+}],$$

here *H*_*NH*_ = *T*_1_, ${V}_{+}={{\rm{\Omega }}}_{a}|{e}_{3}\rangle \langle {0}_{3}|+{{\rm{\Omega }}}_{b}|{e}_{3}\rangle \langle {1}_{3}|$, and ${V}_{-}={V}_{+}^{\dagger }$. Applying the above equations to our setup, we first give the inverse matrix of *T*_1_

$${T}_{1}^{-1}=(\begin{array}{ccccc}\frac{{g}^{2}-2\sqrt{2}J{\rm{\Delta }}}{4{J}^{2}{\rm{\Delta }}} & -\frac{{g}^{2}}{4{J}^{2}{\rm{\Delta }}} & \frac{g}{2J\Delta } & 0 & 0\\ -\frac{{g}^{2}}{4{J}^{2}{\rm{\Delta }}} & \frac{{g}^{2}+2\sqrt{2}J{\rm{\Delta }}}{4{J}^{2}{\rm{\Delta }}} & -\frac{g}{2J{\rm{\Delta }}} & 0 & 0\\ \frac{g}{2J{\rm{\Delta }}} & -\frac{g}{2J{\rm{\Delta }}} & \frac{1}{{\rm{\Delta }}} & 0 & 0\\ 0 & 0 & 0 & 0 & \frac{1}{g}\\ 0 & 0 & 0 & \frac{1}{g} & -\frac{{\rm{\Delta }}}{{g}^{2}}\end{array}).$$

Then the effective Hamiltonian in the first subspace reduces to

21 $$\begin{array}{rcl}{H}_{{\rm{eff}}}^{1} & = & -\frac{{{\rm{\Omega }}}_{a}^{2}}{2{\rm{\Delta }}}\mathrm{|010}\rangle \langle \mathrm{010|}-\frac{{{\rm{\Omega }}}_{b}^{2}}{2{\rm{\Delta }}}\mathrm{|011}\rangle \langle \mathrm{011|}\\  &  & -\frac{{{\rm{\Omega }}}_{a}{{\rm{\Omega }}}_{b}}{2{\rm{\Delta }}}\mathrm{(|011}\rangle \langle \mathrm{010|}+\mathrm{|010}\rangle \langle \mathrm{011|)}.\end{array}$$

### The effective Hamiltonian in the second subspace

The inverse matrix of *T*_2_ in Eq. (9) is

$${T}_{2}^{-1}=(\begin{array}{cccccc}-\frac{{\rm{\Delta }}}{{\eta }_{+}} & \frac{g}{{\eta }_{+}} & 0 & 0 & 0 & 0\\ \frac{g}{{\eta }_{+}} & \frac{\sqrt{2}J}{{\eta }_{+}} & 0 & 0 & 0 & 0\\ 0 & 0 & -\frac{{\rm{\Delta }}}{{\eta }_{-}} & \frac{g}{{\eta }_{-}} & 0 & 0\\ 0 & 0 & \frac{g}{{\eta }_{-}} & -\frac{\sqrt{2}J}{{\eta }_{-}} & 0 & 0\\ 0 & 0 & 0 & 0 & -\frac{{\rm{\Delta }}}{{g}^{2}} & \frac{1}{g}\\ 0 & 0 & 0 & 0 & \frac{1}{g} & 0\end{array}),$$

where ${\eta }_{\pm }={g}^{2}\pm \sqrt{2}J{\rm{\Delta }}$. Based on Eq. (20), the effective Hamiltonian in the second subspace is given by

22 $$\begin{array}{rcl}{H}_{{\rm{eff}}}^{2} & = & \frac{1}{\frac{{g}^{4}}{{J}^{2}{\rm{\Delta }}}-2{\rm{\Delta }}}[{{\rm{\Omega }}}_{a}^{2}\mathrm{|000}\rangle \langle \mathrm{000|}+{{\rm{\Omega }}}_{b}^{2}\mathrm{|001}\rangle \langle \mathrm{001|}\\  &  & +{{\rm{\Omega }}}_{a}{{\rm{\Omega }}}_{b}\mathrm{(|000}\rangle \langle \mathrm{001|}+\mathrm{|100}\rangle \langle \mathrm{000|)]}.\end{array}$$

Based on the above condition $\{|{{\rm{\Omega }}}_{a}|,|{{\rm{\Omega }}}_{b}|\}\ll \{J,g,{\rm{\Delta }}\}$ and $J\ll g$, this effective Hamiltonian ${H}_{{\rm{eff}}}^{2}\approx 0$.

### The effective Hamiltonian in the third subspace

The inverse matrix of *T*_3_ in Eq. (12) is

$${T}_{3}^{-1}=(\begin{array}{ccccc}\sqrt{2}{\alpha }_{-}-2{\beta }^{2}{\rm{\Delta }} & 2{\beta }^{2}{\rm{\Delta }} & {\alpha }_{-} & \beta  & \beta \\ 2{\beta }^{2}{\rm{\Delta }} & \sqrt{2}{\alpha }_{+}-2{\beta }^{2}{\rm{\Delta }} & {\alpha }_{+} & \beta  & \beta \\ {\alpha }_{-} & {\alpha }_{+} & 2{\gamma }_{-}{\gamma }_{+}{\rm{\Delta }} & {\gamma }_{-} & {\gamma }_{+}\\ \beta  & \beta  & {\gamma }_{-} & \frac{1}{2{\rm{\Delta }}} & \frac{1}{2{\rm{\Delta }}}\\ \beta  & \beta  & {\gamma }_{+} & \frac{1}{2{\rm{\Delta }}} & \frac{1}{2{\rm{\Delta }}}\end{array}),$$

with ${\alpha }_{\pm }=\frac{\sqrt{2}{g}^{2}\pm 2J{\rm{\Delta }}}{4{J}^{2}{\rm{\Delta }}}$, $\beta =-\,\frac{g}{2\sqrt{2}J{\rm{\Delta }}}$, and ${\gamma }_{\pm }=\frac{-{g}^{2}\pm 2J{\rm{\Delta }}}{2Jg{\rm{\Delta }}}$. Based on Eq. (20), the effective Hamiltonian in the third subspace can be evaluated explicitly

23 $${H}_{{\rm{eff}}}^{3}=0.$$

### The effective Hamiltonian in the fourth subspace

The inverse matrix of *T*_4_ in Eq. (15) is

$${T}_{4}^{-1}=(\begin{array}{cccc}-\frac{1}{\sqrt{2}J} & 0 & -\frac{1}{2J} & 0\\ 0 & \frac{1}{\sqrt{2}J} & \frac{1}{2J} & 0\\ -\frac{1}{2J} & \frac{1}{2J} & -\frac{2{\rm{\Delta }}}{{g}^{2}} & -\frac{\sqrt{2}}{g}\\ 0 & 0 & -\frac{\sqrt{2}}{g} & 0\end{array}).$$

Based on Eq. (20), the effective Hamiltonian in the fourth subspace can be evaluated explicitly

24 $${H}_{{\rm{eff}}}^{4}=0.$$

## References

[1] Shor PW (1997). Polynomial-time algorithms for prime factorization and discrete logarithms on a quantum computer. SIAM J. Comput..

[2] Feynman RP (1982). Simulating physics with computers. Int. J. Theor. Phys..

[3] Hallgren S (2007). Polynomial-time quantum algorithms for Pell’s equation and the principal ideal problem. J. ACM.

[4] Freedman MH, Kitaev A, Wang Z (2002). Simulation of Topological Field Theories¶ by Quantum Computers. Commun. Math. Phys..

[5] Childs AM (2003). Proceedings of the 35th ACM Symposium on the Theory of Computing.

[6] Sleator T, Weinfurter H (1995). Realizable universal quantum logic gates. Phys. Rev. Lett..

[7] Barenco A (1995). Elementary gates for quantum computation. Phys. Rev. A.

[8] Wu HZ, Yang ZB, Zheng SB (2008). Entanglement-assisted quantum logic gates for two remote qubits. Phys. Lett. A.

[9] Wang H-F, Zhu A-D, Zhang S, Yeon K-H (2013). Optically controlled phase gate and teleportation of a controlled-not gate for spin qubits in a quantum-dot-icrocavity coupled system. Phys. Rev. A.

[10] Wang H-F, Wen JJ, Zhu A-D, Zhang S, Yeon K-H (2013). Deterministic CNOT gate and entanglement swapping for photonic qubits using a quantum-dot spin in a double-sided optical microcavity. Phys. Lett. A.

[11] Wang H-F, Zhu A-D, Zhang S, Yeon K-H (2013). Deterministic CNOT gate and entanglement swapping for photonic qubits using a quantum-dot spin in a double-sided optical microcavity. Phys. Lett. A.

[12] Wang D, Ye L (2014). Proposal for Remotely Realizing Multi-qubit Controlled-Phase Gates. Int. J. Theor. Phys..

[13] Chen Y-H, Xia Y, Chen Q-Q, Song J (2015). Fast and noise-resistant implementation of quantum phase gates and creation of quantum entangled states. Phys. Rev. A.

[14] Xue Z-Y, Zhou J, Chu Y-M, Hu Y (2016). Nonadiabatic holonomic quantum computation with all-resonant control. Phys. Rev. A.

[15] Xue Z-Y (2017). Nonadiabatic Holonomic Quantum Computation with Dressed-State Qubits. Phys. Rev. Applied.

[16] Toffoli, T. Automata, Languages and Programming: Seventh Colloquium, edited by de Bakker, J. W. & van Leeuwen, J. Lectures Notes in Computer Science, Vol. 84 (Springer, New York) (1980).

[17] Jones NC (2012). Layered Architecture for Quantum Computing. Phys. Rev. X.

[18] Clark CR, Metodi TS, Gasster SD, Brown KR (2009). Resource requirements for fault-tolerant quantum simulation: The ground state of the transverse Ising model. Phys. Rev. A.

[19] Jones NC (2012). Faster quantum chemistry simulation on fault-tolerant quantum computers. New J. Phys..

[20] Cory DG (1998). Experimental Quantum Error Correction. Phys. Rev. Lett..

[21] Knill E, Laflamme R, Martinez R, Negrevergne C (2001). Benchmarking Quantum Computers: The Five-Qubit Error Correcting Code. Phys. Rev. Lett..

[22] Chiaverini J (2004). Realization of quantum error correction. Nature (London).

[23] Pittman TB, Jacobs BC, Franson JD (2005). Demonstration of quantum error correction using linear optics. Phys. Rev. A.

[24] Aoki T (2009). Quantum error correction beyond qubits. Nature Phys..

[25] Smolin JA, DiVincenzo DP (1996). Five two-bit quantum gates are sufficient to implement the quantum Fredkin gate. Phys. Rev. A.

[26] Fiurášek J (2006). Linear-optics quantum Toffoli and Fredkin gates. Phys. Rev. A.

[27] DiCarlo L (2009). Demonstration of two-qubit algorithms with a superconducting quantum processor. Nature (London).

[28] Fedorov A, Steffen L, Baur M, Silva MPda, Wallraff A (2012). Implementation of a Toffoli gate with superconducting circuits. Nature (London).

[29] Hua M, Tao MJ, Deng FG (2015). Fast universal quantum gates on microwave photons with all-resonance operations in circuit QED. Sci. Rep..

[30] Wei HR, Deng FG (2014). Universal quantum gates on electron-spin qubits with quantum dots inside single-side optical microcavities. Opt. Express.

[31] Monz T (2009). Realization of the quantum Toffoli gate with trapped ions. Phys. Rev. Lett..

[32] Wei HR, Deng FG (2013). Compact quantum gates on electron-spin qubits assisted by diamond nitrogen-vacancy centers inside cavities. Phys. Rev. A.

[33] Angelakis DG, Santos MF, Bose S (2007). Photon-blockade-induced Mott transitions and XY spin models in coupled cavity arrays. Phys. Rev. A.

[34] Cho J, Angelakis DG, Bose S (2008). Fractional quantum Hall state in coupled cavities. Phys. Rev. Lett..

[35] Irish EK, Ogden CD, Kim MS (2008). Polaritonic characteristics of insulator and superfluid states in a coupled-cavity array. Phys. Rev. A.

[36] Hartmann, M. H., Brandão, F. G. S. & Plenio, M. B. Quantum many-body phenomena in coupled cavity arrays. *Laser Photon*. *Rev*. **2**, 527, and reference therein (2008).

[37] Zheng S-B (2012). Universal quantum logic gates in decoherence-free subspace with atoms trapped in distant cavities. Sci China-Phys Mech Astron.

[38] Shao X-Q, Zheng T-Y, Feng X-L, Oh CH, Zhang S (2014). One-step implementation of the genuine Fredkin gate in high-Q coupled three-cavity arrays. Journal of the Optical Society of America B.

[39] Song L-C, Xia Y, Song J (2014). Noise resistance of Toffoli gate in an array of coupled cavities. Journal of Modern Optics.

[40] Wang H-F, Zhu A-D, Zhang S (2014). One-step implementation of a multiqubit phase gate with one control qubit and multiple target qubits in coupled cavities. Optics Letters.

[41] Xing Y (2017). Spontaneous *PT*-symmetry breaking in non-Hermitian coupled-cavity array. Phys. Rev. A.

[42] Zheng S-B (2013). Implementation of Toffoli gates with a single asymmetric Heisenberg interaction. Phys. Rev. A.

[43] Osnaghi S (2001). Coherent Control of an Atomic Collision in a Cavity. Phys. Rev. Lett..

[44] Kastoryano MJ, Reiter F, Søensen AS (2011). Dissipative preparation of entanglement in optical cavities. Phys. Rev. Lett..

[45] Scully MO, Zubairy MS (1997). Quantum Optics.

[46] Song BS, Noda S, Asano T, Akahane Y (2005). Ultra-high-Q photonic double-heterostructure nanocavity. Nature Mater..

[47] Spillane SM (2005). Ultrahigh-Q toroidal microresonators for cavity quantum electrodynamics. Phys. Rev. A.

[48] Wallraff A (2004). Strong coupling of a single photon to a superconducting qubit using circuit quantum electrodynamics. Nature.

[49] Notomi M, Kuramochi E, Tanabe T (2008). Large-scale arrays of ultrahigh-Q coupled nanocavities. Nat. Photonics.

